# Identification of small segmental translocations in patients with repeated implantation failure and recurrent miscarriage using next generation sequencing after in vitro fertilization/intracytoplasmic sperm injection

**DOI:** 10.1186/s13039-015-0207-7

**Published:** 2015-12-30

**Authors:** Jian Ou, Wei Wang, Tao Feng, Lianming Liao, Qingxia Meng, Qinyan Zou, Jie Ding, Aiyan Zheng, Chengying Duan, Peipei Li, Qiang Liu, Chunhua Lin, Hong Li

**Affiliations:** Center of reproduction and genetics Suzhou Municipal Hospital, Suzhou, Jiangsu P.R.China; Peking Jabrehoo Med Tech., Ltd, Beijing, P.R.China; Central Laboratory, The Union Hospital of Fujian Medical University, Fuzhou, Fujian P.R.China

**Keywords:** NGS, Array CGH, PGS, RIF, RM, Translocation

## Abstract

**Background:**

To develop a novel preimplantation genetic screening (PGS) test using next generation sequencing(NGS) as a alternative to current array comparative genomic hybridization (array CGH) method for detection of small segmental translocations in two patients with repeated implantation failure (RIF) and recurrent miscarriage (RM). Inconsistent results were resolved by validation with fluorescence in situ hybridization (FISH).

**Case Presentation:**

One couple with normal cytogenetic and array CGH result suffered from implantation failure. Later NGS analysis showed 46,XY.ngs[GRCh37/hg19] 9p24.3-9p24.1(10,291-8,680,890×1),13q33.1-13q34(103,046,327-114,785,444×3). The other couple with normal cytogenetic and array CGH result also received NGS analysis. Due to the detected abnormal finding, which was 46,XY.ngs 4q34.3-4q35.2(179,673,982-191,016,503×3),6p25.3-6p22.3 (146,672-17,829,693×1), the couple decided against the corresponding embryo transfer.

**Conclusions:**

The NGS approach is a reliable alternative to array CGH for the discovery of small segmental translocations in patients with RIF and RM.

## Background

Repeated implantation failure (RIF) usually refers to failure of transferred embryos to implant following at least three in vitro fertilization (IVF) treatment cycles, in which 1–2 embryos of high grade quality are transferred in each cycle [1, 2]. Abnormal embryo, endometrium or immune system will result in implantation failure. Therefore, in assessing RIF, the embryo should be evaluated for potential abnormality [3]. Recurrent miscarriage (RM) is defined as two or more miscarriage [4]. Etiologic factors can be identified in approximately 50 % of these couples. Preimplantation genetic screening (PGS) was proposed for couples with unexplained RM and RIF because aneuploidy of the embryo may cause RM and RIF [5–7].

Currently comparative genomic hybridization (array CGH) techniques have been used for PGS to analyzing metaphase II oocytes and their polar bodies, cleavage stage embryos, and blastocysts [8, 9]. PGS by array CGH with single euploid blastocyst transfer has been successfully applied in patients with RIF [10]. However, small segmental structure abnormalities of the chromosomes are sometimes not picked up in the currently available commercial array CGH due to insufficient resolution [11, 12], degraded DNA from apoptosis [13], whole genome amplification (WGA) bias [14] and no specific chromosomal abnormalities detected before induction on PGS.

Next generation sequencing (NGS) was introduced for PGS recently [15]. In retrospective and blind validation studies, a high concordance between NGS and array CGH for the same WGA products has been demonstrated on aneuploidy screening. What is more, NGS can correct the potential bias caused by WGA and is therefore more accurate in detecting chromosomal structural abnormality of small fragments [16]. Here, we used NGS to reanalyze the biopsied samples with normal result of array CGH analysis in two patients with RIF and RM after PGS. To the best of our knowledge, this is the first report of abnormal NGS results in RIF and RM patients with normal array CGH results PGS.

## Methods

### Case presentation

#### Case 1

The couple has suffered spontaneous abortion for three times from 2008 to 2009. Giemsa (G) banding karyotyping of 400 bands showed no obvious structural abnormality for both. In vitro fertilization and embryo transfer (IVF-ET) treatment was done in our hospital in July 2012. The first fresh cycle transplantation with two embryos did not achieve pregnancy. Then frozen embryo recovery transplantation (FET) was performed with two embryos in December 2012. Spontaneous abortion occurred after 7 weeks’ pregnancy. In August 2013, FET was performed again. However, no blastocyst was formed after frozen embryos were cultured. In September 2013, array CGH PGS was used for screening embryos. One normal embryo was transferred in July 2014 but the pregnancy test was negative. In February 2015, fifth spontaneous abortion occurred again 70 days after natural conception. Products of fifth abortion were analyzed by using multiple connected probe amplification technology (MLPA) on DNA extracted from fetal tissues. The remaining DNA sample used for PGS by array CGH analysis was also reanalyzed by NGS in May 2015.

#### Case 2

The couple received assisted reproductive technology (ART) treatment in October 2011 because of primary infertility for 5 years. G banding karyotyping of 400 bands showed no obvious structural abnormality of this couple. Two embryos were transplanted in two FET cycles in March and June 2012 respectively without pregnancy. In November 2012, the third FET was performed. Although pregnancy was achieved, spontaneous abortion occurred after 2 months. In November 2013, the forth FET was performed. Against spontaneous abortion occurred after 57 days. In January 2015, PGS by array CGH was performed and two embryos were found to be normal, in May 2015, the NGS was used to analyze the remaining DNA sample used in array CGH.

### Karyotyping

Karyotyping was performed using peripheral blood from the couples. Metaphase chromosomes were investigated by G banding with at least 20 metaphases analyzed for each patient.

### Embryo culture and biopsy

All patients used a long protocol, or a GnRH (Gonadotropin-releasing hormone) antagonist protocol for controlled ovarian hyperstimulation. Oocytes were retrieved 34 to 35 h after hCG injection and fertilized with ICSI as previously described [17]. All embryos were cultured in vitro in 20 ul microdrops of G1 culture medium (Vitrolife), and on day 3 post of fertilization, they were transferred to 20ul microdrops of G2 (Vitrolife) to blastocysts stage under standard incubation conditions. All embryos were cultured to the blastocyst stage and scored according to Gardner’s grading scale on day 5 after fertilization. Zona drilling for blastocyst trophectoderm biopsy was performed on the morning of Day 5 using the ZILOS-tk laser (Hamilton Thorne Biosciences). Blastocysts were incubated for a further 4–6 h to allow blastocoele expansion and extrusion of the trophectoderm cells from the zona drilling hole. Blastocyst biopsy performed within 10ul microdrops of Qunnis Advantage Medium wtih HEPES (SAGE) add with 5 % Human Serum Albumin (SAGE). Suction with the biopsy pipette (Origio) and laser drilling were applied to dissect four to five trophectoderm cells from the blastocysts. The biopsied cells were rinsed three time with G-MOPS (Vitrolife) medium and transfered to RNAse–DNAse-free PCR tubes (Axygen) with the minimum medium.

#### Whole genome amplification (WGA)

The biopsied trophectoderm cells, pooled single cell DNA and negative control were subject to WGA using single cell SurePlex WGA Kit (BlueGnome). This kit included three steps of fragmentation (lysis), library preparation and amplification. This WGA method is based on random fragmentation of genomic DNA and conversion of the resulting small fragments to PCR-amplifiable library molecules flanked by universal priming sites. Amplification products were stored at –20 °C. To avoid contamination, this process should be all handled in a ventilation cabinet.

#### Array comparative genomic hybridization (Array-CGH)

Array-CGH was performed using oligonucleotide-based custom arrays (8 × 60 K) (Agilent Tech), according to the protocol of Agilent oligonucleotide array-based CGH for single cell analysis (Agilent Tech) and scanning on Agilent microarray scanner. Then Agilent feature extraction used for data interpretation and Agilent Genomic Workbench 7.0/CytoGenomics v2.9 used for analysis.

#### Next generation sequencing (NGS)

ION XPRESS LIBRARY kit (Life Tech) was used to construct the sequencing library. A total of 100 ng of WGA products were incubated in the buffer and enzyme mix II (Ion Shear™Plus) at 37 °C for 35 min for fragmenting. DNA was purified by using Agencourt®AMPure®XP beads (BeckMan) and connected with Ion Xpress™Barcode 1(Life Tech). The amplified libraries were run on an E-Gel®SizeSelect™ 2 % agarose gel (Invitrogen) and the fraction corresponding to a 200 bp insert was purified with AMPure beads (Agencourt). All samples were diluted and used as a template for amplification using the Ion OneTouch system (Ion PGMTMTemplate OT2 200 Kit, Life Technologies). Sample enrichment was performed on the Ion OneTouch ES module and Ion 316 chips were used for sequencing. Data were analyzed using the E&S System developed by Peking Jabrehoo Med Tech., Ltd (Beijing, China), providing the percentage of DNA sequence reads mapped to each chromosome. In brief, the method splits chromosome into segments ranging from 600 k to 1 Mb. Then a set of 30 reference values is created by averaging the percentage of mapped reads attributable to each segment in a series of euploid samples. The percentage of reads derived from a given chromosome segment longer than 10 Mb for an embryo (test) sample was divided by the reference value for the same segment. The CNVs larger than 4 megabase (>4 M) would be truly detected actually. Chromosomal duplication were associated with ratios >1.25 and deletion with ratios <0.75 [18].

### Fluorescence in situ hybridization (FISH)

Chromosome analysis was done using a Carl Zeiss AXIO microscope (Germany) with image analysis system (CytoVision Leica). Fluorescence in situ hybridization (FISH) was performed according to the previously published protocols [19]. Commercially available probes (Abbott Molecular) like Tel 4q, Tel 6p, Tel 9p, Tel 9q, LSI 13 and CEP4 were used.

### Multiplex ligation-dependent probe amplification (MLPA)

MLPA was performed using SALSA MLPA P036-E2 kit (MRC-Holland). For each MLPA assay, 100 ng of DNA was used following the manufacturer’s protocols. This P036-E2 Subtelomeres mix 1 probemix contains one MLPA probe for each subtelomeric region and is designed to detect deletions/duplications of these subtelomeric regions.

## Results

### Case 1

**Fig. 1:**
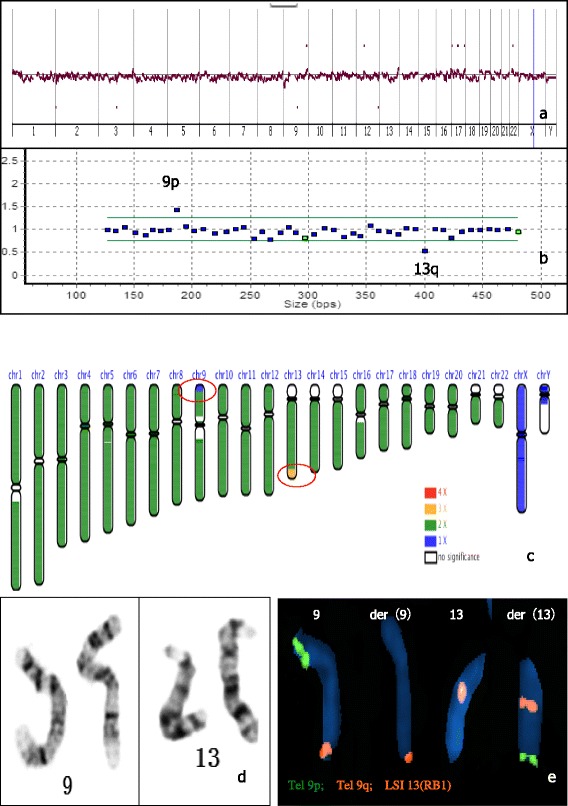
Case 1. 1**a**: PGS profile of array CGH was normal; 1**b**: The MLPA result of the fifth abortion sample was 9p+,13q-; 1**c**: The NGS result was 46,XY.ngs[GRCh37/hg19] 9p24.3-9p24.1(10,291-8,680,890×1),13q33.1-13q34(103,046,327-114,785,444×3) (indicated by red circle); 1**d**: The G banding karyotyping of 550 bands showed no obvious structural abnormality; 1**e**: FISH showed reciprocal translocation in the male chromosome. Tel 9p: green; Tel 9q: orange; LSI 13 (RB1): orange

The patient received transfer of normal embryo as indicated by array CGH PGS (Fig. 1a). However, the pregnancy test was negative. The MLPA result showed that the 9p probe was duplicated, and the 13q probe was deleted in the fifth abortion sample (Fig. 1b). The DNA sample for array CGH assay was reanalyzed by NGS, which revealed 46,XY.ngs [GRCh37/hg19] 9p24.3-9p24.1(10,291-8,680,890×1), 13q33.1-13q34(103,046,327-114,785,444×3) (Fig. 1c). G banding karyotyping of 550 bands also showed no obvious structural abnormality (Fig. 1d). FISH analysis with Tel 9p, Tel 9q and LSI 13 probes confirmed the existence of the reciprocal translocation in the male chromosome as 46,XY,t(9;13)(p24.1;q33.1). (Figure 1e).

### Case 2

**Fig. 2:**
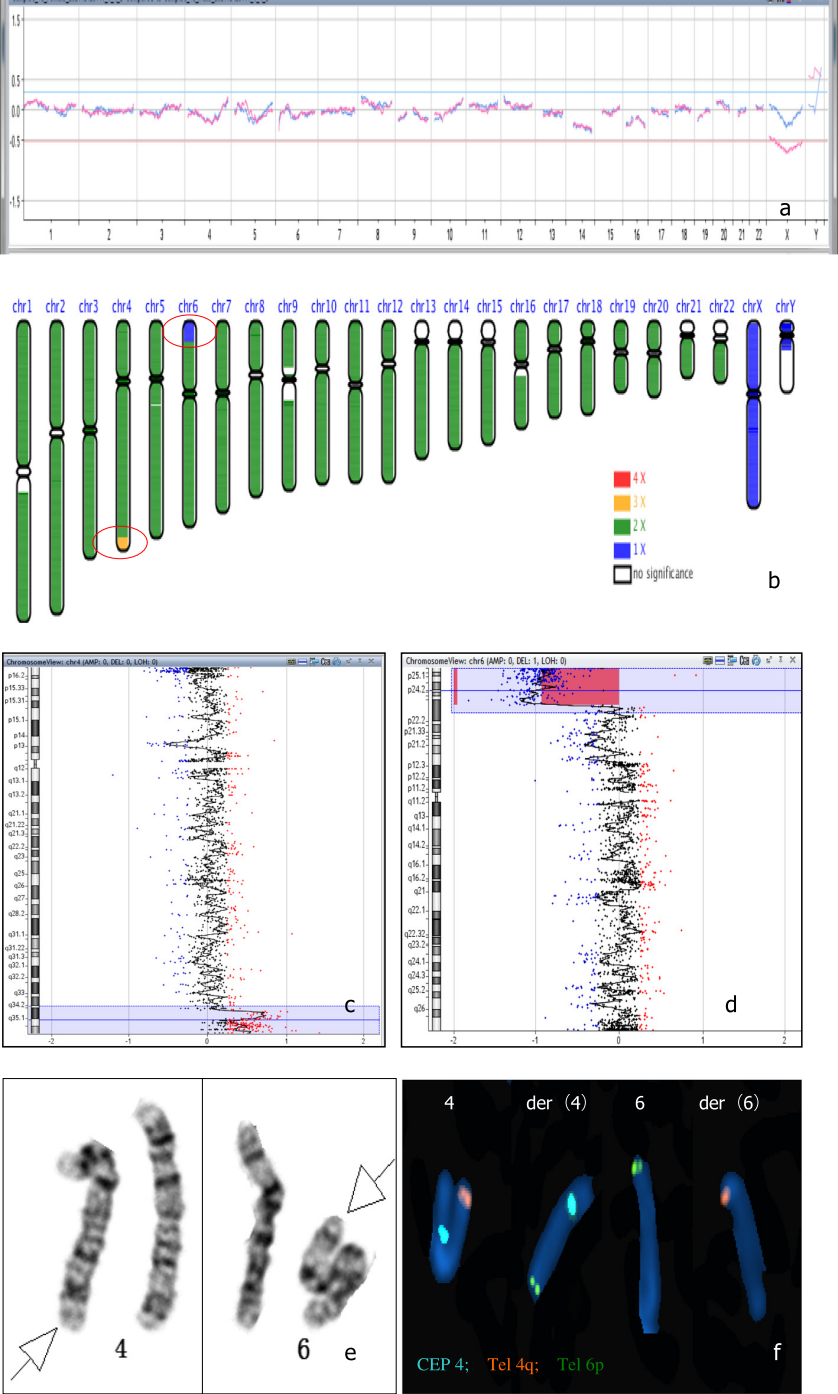
Case 2. 2**a**: PGS profile by array CGH was normal; 2**b**: PGS by NGS was 46,XY.ngs 4q34.3-4q35.2(179,673,982-191,016,503×3), 6p25.3-6p22.3 (146,672-17,829,693×1) (indicated by red circle); 2**c**, 2**d**: The array CGH result of the second abortion sample was arr 4q34.3-q35.2 (179,554,876-190,864,789) × 3, 6p25.3-p22.3(347,038-17,543,199) × 1; 2**e**: The G banding karyotyping of 550 bands showed 46,XX,t(4; 6)(q34.3;p22.3); 2**f**: FISH showed reciprocal translocation of female chromosome. Tel 4q: orange; CEP4: Aqua; Tel 6p: green

The array CGH PGS results showed normal (Fig. 2a) but the NGS result of the same sample was 46,XY.ngs 4q34.3-4q35.2(179,673,982-191,016,503×3), 6p25.3-6p22.3 (146,672-17,829,693×1), (Fig. 2b). To clarify the discrepancy, result of array CGH for the second abortion product was found to be 46,XY.arr 4q34.3-q35.2 (179,554,876-190,864,789) × 3, 6p25.3-p22.3(347,038-17,543,199) × 1 (Fig. 2c,d). Further G banding karyotyping of 550 bands showed 46,XX,t(4;6)(q34.3;p22.3) of the female (Fig. 2e). Tel 4q, CEP4 and Tel 6p three FISH probes further verify the existence of the reciprocal translocation of female chromosome (Fig. 2f) (Table 1).

**Table 1:** Two Cases with inconsistent results between NGS and array CGH, and carrier validation by FISH

Cases	RIF times	RM times	embryo array CGH PGS result	embryo NGS result	carrier FISH
1	2	5	normal	ngs9p24.3-9p24.1(10,291-8,680,890×1)	ish t(9;13)(p24.1;q33.1)
				13q33.1-13q34(103,046,327-114,785,444×3)	(9pter-,9qter+;9pter+,RB1+)
2	2	4	normal	ngs4q34.3-4q35.2(179,673,982-191,016,503×3)	ish t(4;6) (q34.3;p22.3)
				6p25.3-6p22.3 (146,672-17,829,693×1)	(6pter+,cep4+;6pter-,4qter+)

## Discussion

It was found that about 15 % of the RIF patients had chromosome abnormalities, including chromosomal translocation, inversion and mosaic [20]. Most of these structural abnormalities can be revealed by routine karyotyping. Because of the low resolution, many small chromosome structural abnormalities of peripheral blood cells cannot be detected in the routine karyotyping. Indeed, in the present study both couple had not found obvious structural abnormality in routine karyotyping even they had RIF and RM.

In the first case, transfer of normal embryo after array CGH PGS did not result in pregnancy and the MLPA result of the fifth abortion tissues showed abnormal chromosome. NGS was applied to reanalyze the same WGA sample of the embryo and small segmental translocations were revealed. FISH assay of the couple’ peripheral blood cells confirmed there were small segmental translocations in the male. It is concluded that PGS by array CGH failed to detect small segmental translocations, which may be responsible for RIF.

Lesson learned from case 1 urged us to reanalyze embryos of case 2 before transplantation using NGS even array CGH showed euploid. PGS by NGS showed that one embryo was 46,XY.ngs 4q34.3-4q35.2(179,673,982-191,016,503×3), 6p25.3-6p22.3 (146,672-17,829,693×1). The result was confirmed by array CGH analysis of the second abortion sample and FISH of the peripheral blood cells of the couple, which revealed small segmental translocations of the female. We therefore abandoned the embryo to avoid potential miscarriage after embryo transfer.

Genetic test is very important for patients with RIF and RM. Analysis of the abortion tissue samples with more sensitive methods (such as high resolution karyotyping, FISH, MLPA, array CGH and NGS) to identify small abnormal fragments of chromosomes is necessary in addition to the traditional karyotyping. Fiorentino et al reported [21] that the minimal fragment detected by NGS was 14 Mb in PGS. Here we detected an 8.27 Mb abnormal fragment. Whether smaller fragment can be detected deserves further study. In addition, Tan et al reported [11] that among the chromosomally abnormal blastocysts subjected to both NGS and SNP array tests, 7 blastocysts gave inconsistent results, which were further validated by qPCR. All the qPCR signals were in accordance with NGS. Here we found there was discordance (abnormal vs normal) between NGS and array CGH for the same WGA sample for the first time on clinical PGS cycles. As expected, the NGS result was supported by other tests and was therefore considered to be correct.

Any testing has to balance the benefits of identifying euploid embryos with the potential costs. Now the NGS used here can truly detect the small segment larger than 4 megabase (>4 M) and the cost of sequencing was already competitive with array tests, the whole reagent cost of sequencing for WGA sample of the embryo was currently less than $200. We have reasons to believe that eventually the advantages of NGS will be brought to PGS patients and widely used in the clinical practice.

## Conclusions

In summary, sometimes the patients with small segmental translocations actually had not found obvious structural abnormality in routine karyotyping even they had RIF and RM, and the currently available commercial array CGH used for aneuploidy screening in PGS for these patients with RIF and RM sometimes failed to detect small segmental translocations. In our study we found the NGS approach was effective in characterizing small abnormal chromosomal fragments. It was a reliable alternative to array CGH to be used for PGS to avoid failure of detection of small abnormal chromosomal fragments in patients with RIF and RM.

## Consent

A written informed consent was obtained from each couple after counseling for the publication of this report and any accompanying images. The study was approved by the Institutional Review Board. All experimentations were performed according to the Helsinki Declaration.
